# Subclinical pulmonary dysfunction contributes to high altitude pulmonary edema susceptibility in healthy non-mountaineers

**DOI:** 10.1038/s41598-017-14947-z

**Published:** 2017-11-02

**Authors:** Rajinder K. Gupta, Poonam Soree, Koundinya Desiraju, Anurag Agrawal, Shashi Bala Singh

**Affiliations:** 1 Defence Institute of Physiology and Allied Sciences. Timarpur, Delhi, 110054 India; 2 CSIR - Institute of Genomics and Integrative Biology, Mall Road, Delhi, 110007 India

## Abstract

HAPE susceptible (HAPE-S, had HAPE episode in past) subjects may have subclinical cardio-pulmonary dysfunction. We compared the results of pulmonary function tests in 25 healthy HAPE-S non-mountaineers and 19 matched HAPE resistant (HAPE-R, no HAPE episode in past). Acute normobaric hypoxia (FIo_2_ 0.12) was administered at sea level to confirm hypoxia intolerance in HAPE-S. Unlike HAPE-R, HAPE-S subjects had elevated baseline and post-hypoxia systolic pulmonary arterial pressures (20.9 ± 3 vs 27.3 ± 5 mm Hg during normoxia and 26.2 ± 6 vs 45.44 ± 10 mm Hg during hypoxia, HAPE-R vs HAPE-S). Forced vital capacity (FVC) and single breath alveolar volume (SBVA) were significantly lower in HAPE-S compared to HAPE-R (FVC: 4.33 ± 0.5 vs 4.6 ± 0.4; SBVA: 5.17 ± 1 vs 5.6 ± 1 Lt; HAPE-S vs HAPE-R). Two subgroups with abnormal pulmonary function could be identified within HAPE-S; HAPE-S1 (n = 4) showed DLCO>140% of predicted, suggestive of asthma and HAPE-S2 (n = 12) showed restrictive pattern. Each of these patterns have previously been linked to early small airway disease and may additionally represent a lower cross-sectional area of the pulmonary vascular bed, related to lower lung volumes. HAPE susceptibility in healthy non-mountaineers may be related to sub-clinical pulmonary pathology that limits compensatory rise in ventilation and pulmonary circulation during hypoxic stress.

## Introduction

High Altitude Pulmonary Edema (HAPE) is a hydrostatic oedema which occurs at altitude more than 2500 m in susceptible individuals^1–3^. The risk is believed to be multifactorial with prior work from our and other labs implicating genetic abnormalities in the hypoxic response pathway as well as chronically up regulated hypoxic response^4–7^. Subclinical cardiopulmonary dysfunction may have a synergistic role in this context and it has been previously reported that HAPE resistant (HAPE-R) subjects have a significantly greater forced vital capacity (FVC), on spirometry, than HAPE susceptible (HAPE-S)^8,9^. Low FVC volumes have been reported to be one of the strongest non-invasive predictors of cardiopulmonary risk^10–12^. While the mechanism for this remains unknown, it is thought that the underlying chronic low-grade inflammation have a significant role in causing reduced lung function^13,14^. To determine the possible role of pulmonary function testing, especially FVC, for predicting HAPE-risk, we conducted this study in a non mountaineer population that was physically fit and carefully followed a prescribed acclimatisation schedule during ascent to high altitude. We measured pulmonary functions in both HAPE-S and HAPE-R followed by acute normobaric hypoxia stress given to all individuals at sea level and pulmonary vascular responses were recorded to confirm hypoxia intolerance in HAPE-S.

## Methods

We studied 44 male Army soldiers whose susceptibility and resistance for HAPE was known from their previous stay at high altitude. All participants were non smokers, lowlanders, free of airway infection and receiving no medication at the time of study. None of the subject has resided above 2000 m within last six months before the baseline measurements were carried out in Delhi, India at an altitude 293 m above sea level. The HAPE susceptible subjects (HAPE-S, n = 25) suffered the illness which was confirmed radiologically in spite of observing acclimatisation schedule. HAPE resistant (HAPE-R) subjects were drawn from colleagues who were deployed similarly but did not have any adverse event and showed normal pulmonary vascular response^15^ at sea level (sPpa <38 mm Hg at Fio2 = 0.12). Two groups were screened out of twenty-five HAPE-S subjects based on abnormal pulmonary functions: a) DLCO>140% predicted^16^ for inclusion under HAPE-S1, n = 4. b) FVC and TLC < average normal level of % predicted for inclusion under HAPE-S2, n = 12. We selected 140% of the predicted DLCO as the cutoff for the selection of subjects under HAPE-S1 group for the following reasons: 1) this is well outside the common range of inter subject variability and (2) this increase in DLCO would be unlikely to be secondary to technical or physiological variations during testing. All experimental protocols were approved by Defence Institute of Physiology and Allied Sciences Ethics Committee for scientific experiments. Informed written consent was obtained from all participants before enrolment in the study. All methods were carried out in accordance with the approved guidelines and regulations.

### General Procedures

The subjects were investigated in the supine position while breathing synthetic gas mixtures consisting of 21 or 12% oxygen (FiO2 = 0.12) mixed in nitrogen. The hypoxic gas mixture corresponded to an altitude of 4500 m. Inhalation was performed via a tight fit face mask. Systolic pulmonary artery pressure (sPpa) was recorded before and at the end of 30 min. of hypoxic stress.

### Determination of pulmonary artery systolic pressure

Pulmonary^17^ artery hemodynamics was measured non invasively using echocardiography. Echocardiography studies were performed with My Lab 30 Gold Line ultrasonograph (Esaote India). Standard parasternal and apical two dimensional views were obtained, and color flow directed pulse wave Doppler measurements of transvalvular flows and continuous wave Doppler measurements of tricuspid regurgitant flow were obtained. A single lead electrocardiogram was recorded on the ultrasonograph. Measures obtained using this noninvasive technique correlates closely with those obtained using cardiac catheterisation.

Pulmonary artery systolic pressure (sPpa) was calculated as follows

$${\rm{sPpa}}=[4{({{\rm{TR}}}_{{\rm{vel}}})}^{2}]+{\rm{RAP}}$$

where TR_vel_ is tricuspid regurgitation jet velocity and RAP is the estimated right atrial pressure based on respiration variation in inferior venacava size.

### Pulmonary function measurement

Spirometry and DLCO measurements were performed in compliance with American Thoracic Society (ATS) guidelines^18–20^. Basal pulmonary function data was measured using dry, rolling seal spirometer (P. K. Morgan, Kent, UK). In addition to lung volumes and flow measurements, functional residual capacity (FRC) was measured by the closed circuit Helium dilution test. Diffusion capacity of the lung (DLCO) was determined by single-breath, breath-holding technique. The DLCO was routinely adjusted for hemoglobin if value was outside the normal range. Reference values for predicted DLCO were from North Indian population^21,22^. The best value from three attempts were recorded as both absolute values and as percentage of the predicted values, based on age and body weight.

### Quality control

DLCO tests were performed by trained technician using equipment that was calibrated using a 3 litre syringe. Subjects performed at least one repeat DLCO test after the initial manure. If DLCO and SBVA values were not within 10%, the test was repeated a third time, and the values that were the closest match were used.

### Statistics

Difference between two groups was assessed by t-test after confirming normal distribution. Since the values are highly auto-correlated, no further correction was performed. Analysis was done using R statistical programming language. All data are presented as means  ± SD. P < 0.05 is considered significant.

## Results

HAPE-S showed abnormal pulmonary vascular response to hypoxia. Table 1 shows anthropometric data, pulmonary functions and pulmonary vascular response to hypoxic challenge for HAPE-S and HAPE-R subjects. The two groups were similar in terms of age, height and weight. Hb was normal in both groups but slightly higher in HAPE-R. Baseline systolic pulmonary artery pressure (sPpa) was high and showed exaggerated response to acute hypoxia in HAPE-S.

**Table 1:** Baseline anthropometery, pulmonary functions and pulmonary vascular response to hypoxia of all HAPE susceptible subjects (n = 25).

	HAPE-S (n = 25)	HAPE-R (n = 19)	p value
Age (yrs)	31.8 ± 6.2	28.8 ± 6	0.060
Height (cms)	170.2 ± 5	171.5 ± 8	0.183
Weight (Kg)	72.6 ± 7	71 ± 8	0.246
BMI (%)	25.12 ± 3	24.2 ± 3	0.133
Hb (gm%)	14.35 ± 1	15.5 ± 1*	0.004
FVC (L)	4.33 ± 0.5	4.6 ± 0.4*	0.020
FVC (%PRE)	93.8 ± 12	95.9 ± 9	0.250
FVC (AvgPred)	4.63 ± 1	4.8 ± 0.5	0.103
FEV1(Lt)	3.55 ± 0.5	3.8 ± 0.3*	0.028
FEV1%	91.88 ± 13	95 ± 9	0.381
FEV1/FVC	82 ± 7	83.9 ± 5	0.156
FEV1/FVC%	97.4 ± 9	96.7 ± 6	0.373
FEF25–75%	91.08 ± 26	93.1 ± 21	0.787
FRC(L)	3.24 ± 1	3.3 ± 1	0.344
FRC%	106.44 ± 18	107.9 ± 26	0.414
RV(L)	1.74 ± 0.45	1.6 ± 1	0.161
RV%	116.7 ± 31.4	107.9 ± 34	0.189
TLC(L)	6.16 ± 1	6.3 ± 1	0.262
TLC%	100.68 ± 13	99.9 ± 12	0.425
TLC (AvgPred)	6.12 ± 1	6.3 ± 0.5	0.145
DLCO ml/min/mm Hg	34.8 ± 7	36.9 ± 5	0.141
DLCO%	110.1 ± 22	113.8 ± 17	0.277
SBVA(L)	5.17 ± 1	5.6 ± 1*	0.012
SBVA%	72.8 ± 8	77.4 ± 6*	0.026
DLCO/SBVA	6.7 ± 1	6.6 ± 1	0.395
DLCO/SBVA%	125.1 ± 18	121.6 ± 15	0.253
sPpa(normoxia)	27.3 ± 5	20.9 ± 3*	0.000
sPpa (hypoxia)	45.4 ± 10	26.2 ± 6*	0.000

FVC-forced vital capacity, FEV1-Forced expiratory volume in 1 sec, FRC-functional residual capacity, RV-residual volume, TLC-total lung capacity, DLCO-pulmonary diffusion capacity for carbon monoxide, SBVA-Single breath alveolar volume, sPpa - systolic pulmonary artery pressure. *p < 0.05-statistically significant.

HAPE-S showed low lung volumes. Pulmonary function data (Table 1) shows significantly lower FVC, FEV1 and SBVA in HAPE-S compared to HAPE-R. To determine how many of the otherwise healthy HAPE-S subjects would be defined as clinically abnormal using standard PFT definitions, we used predicted values for each subject and classified each subject, using standard clinical guidelines (see methods). All HAPE-R subjects and nine of twenty-five HAPE-S subjects had normal PFT. In the sixteen HAPE-S subjects with at least one abnormal PFT result, two types of abnormal patterns emerged —abnormally high DLCO (HAPE-S1) or a restrictive pattern in (HAPE-S2), as shown in Tables 2 and 3.

**Table 2:** Baseline anthropometry, FVC (forced vital capacity) and FEV1 (Forced expiratory volume in 1 sec), values in HAPE-S1, HAPE-S2 and HAPE-R.

	HAPE-S1(n = 4)	HAPE-S2 (n = 12)	HAPE-R (n = 19)
Age (years)	29 ± 4.55	31.23 ± 6.29	28.8 ± 5.6
Height (cm)	168.7 ± 7.14	171.5 ± 4.8	171.5 ± 4
Weight (Kg)	70.25 ± 2.63	71.33 ± 7.16	71 ± 8.1
BMI (Kg/cm^2^)	24.8 ± 2.58	24.3 ± 3.19	24.2 ± 2.79
Hb (gm/dl)	14.8 ± 1.15	14.52 ± 1.5	15.5 ± 1
FVC (Lt)	4.66 ± 0.3	4.05 ± 0.46^a^	4.6 ± 0.3^b^
FVC%	101.7 ± 10.05	84.83 ± 7.15^a^	95.9 ± 8.5^b^
FEV1(lt)	3.92 ± 0.48	3.34 ± 0.49^a^	3.8 ± 0.3^b^
FEV1/FVC (Lt)	83.7 ± 5.68	82.5 ± 7.9	83.9 ± 5
FEV1/FVC%	99.25 ± 6.7	97.8 ± 10.03	96.7 ± 6.1

Values are presented as Mean ± SD; ^a^p < 0.05 (HAPE-S1 versus HAPE-S2); ^b^p < 0.05 (HAPE-S2 versus HAPE-R).

**Table 3:** TLC (Total lung capacity), FRC (Functional residual capacity), DLCO (pulmonary diffusion capacity for carbon monoxide), SBVA (Single breath alveolar ventilation) and DLCO/SBVA values in HAPE-S1, HAPE-S2 and HAPE-R.

	HAPE-S1(n = 4)	HAPE-S2(n = 12)	HAPR-R(n = 19)
TLC(Lt)	6.56 ± 0.52	5.73 ± 0.57^a^	6.3 ± 0.6^b^
TLC%	110.25 ± 18.7	90.9 ± 6.14^a^	99.9 ± 12.28^b^
FRC (Lt)	3.69 ± 0.53	3 ± 0.62	3.3 ± 0.7
FRC%	125.7 ± 20.37	96 ± 16.03	107.7 ± 25.9
DLCO ml/kg/mm Hg	46.7 ± 3.24	31.57 ± 5.58^a^	36.9 ± 4.9^b,c^
DLCO%	147.5 ± 6.6	97.92 ± 16.47^a^	113.8 ± 16.7^b,c^
SBVA (Lt)	5.79 ± 0.3	4.8 ± 0.53^a^	5.6 ± 0.53^b^
SBVA%	83.25 ± 4.19	67.17 ± 5.6^a^	77.4 ± 6.3^b^
DLCO/SBVA	8.07 ± 0.28	6.49 ± 0.99^a^	6.6 ± 0.7^c^
DLCO/SBVA%	147.5 ± 2.65	120.75 ± 20.2^a^	121.6 ± 15.1^c^

Values are presented as Mean ± SD. ^a^p < 0.05 (HAPE-S1 versus HAPE-S2); ^b^p < 0.05 (HAPE-S2 versus HAPE-R); ^c^p < 0.05 (HAPE-S1 versus HAPE-R).

**Figure 1:**
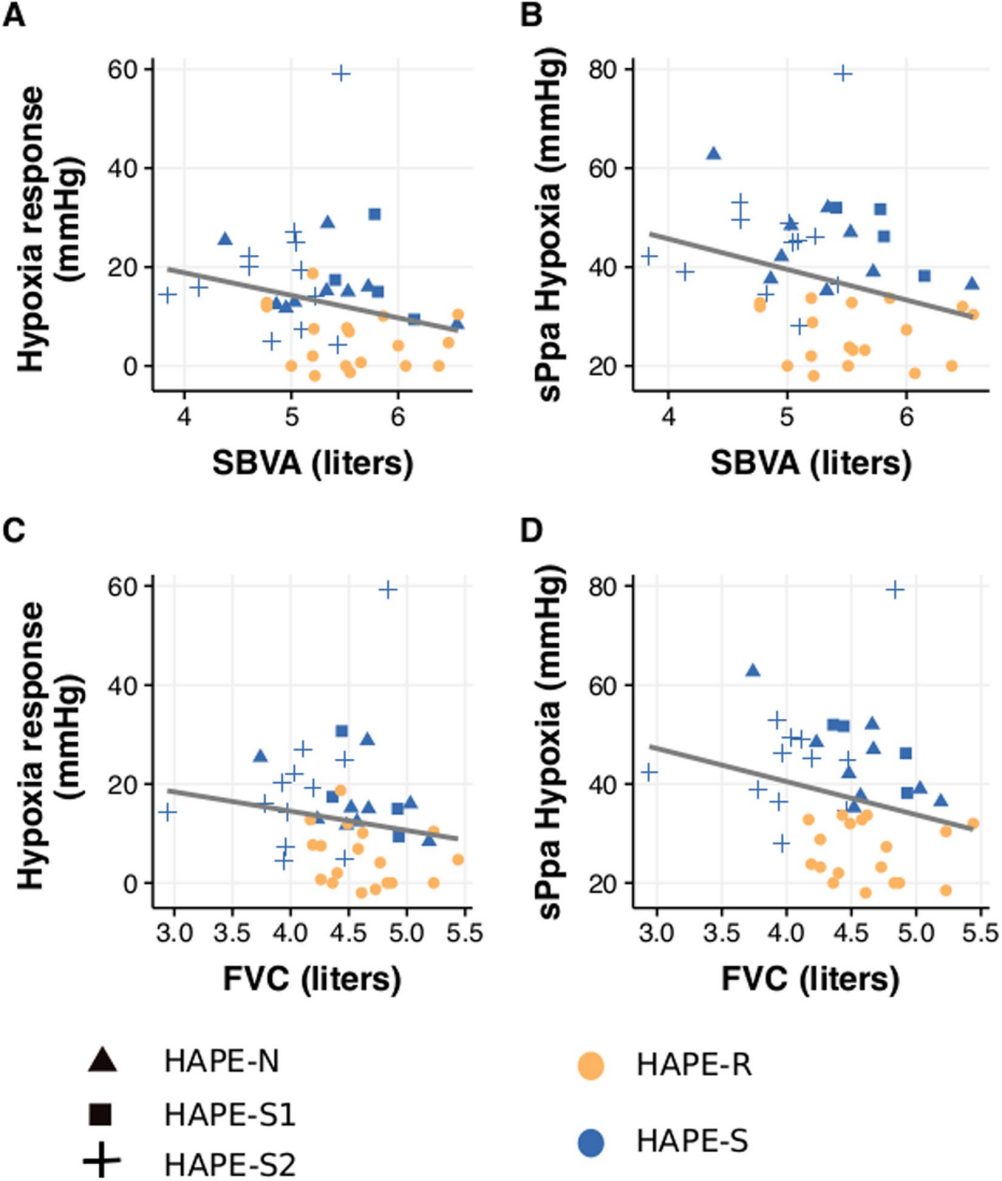
Scatter plots showing individual data of different HAPE-S subgroups: HAPE-S1, HAPE-S2, HAPE-N (HAPE-S with normal PFT) and HAPE-R individuals. Plots between Hypoxic Response with single breath alveolar volume (SBVA) (panel A) and forced vital capacity (FVC) (panel C) and Systolic pulmonary artery pressure (sPpa) in hypoxic condition with SBVA (panel B) and FVC (panel D) showing negative correlation.

**Figure 2:**
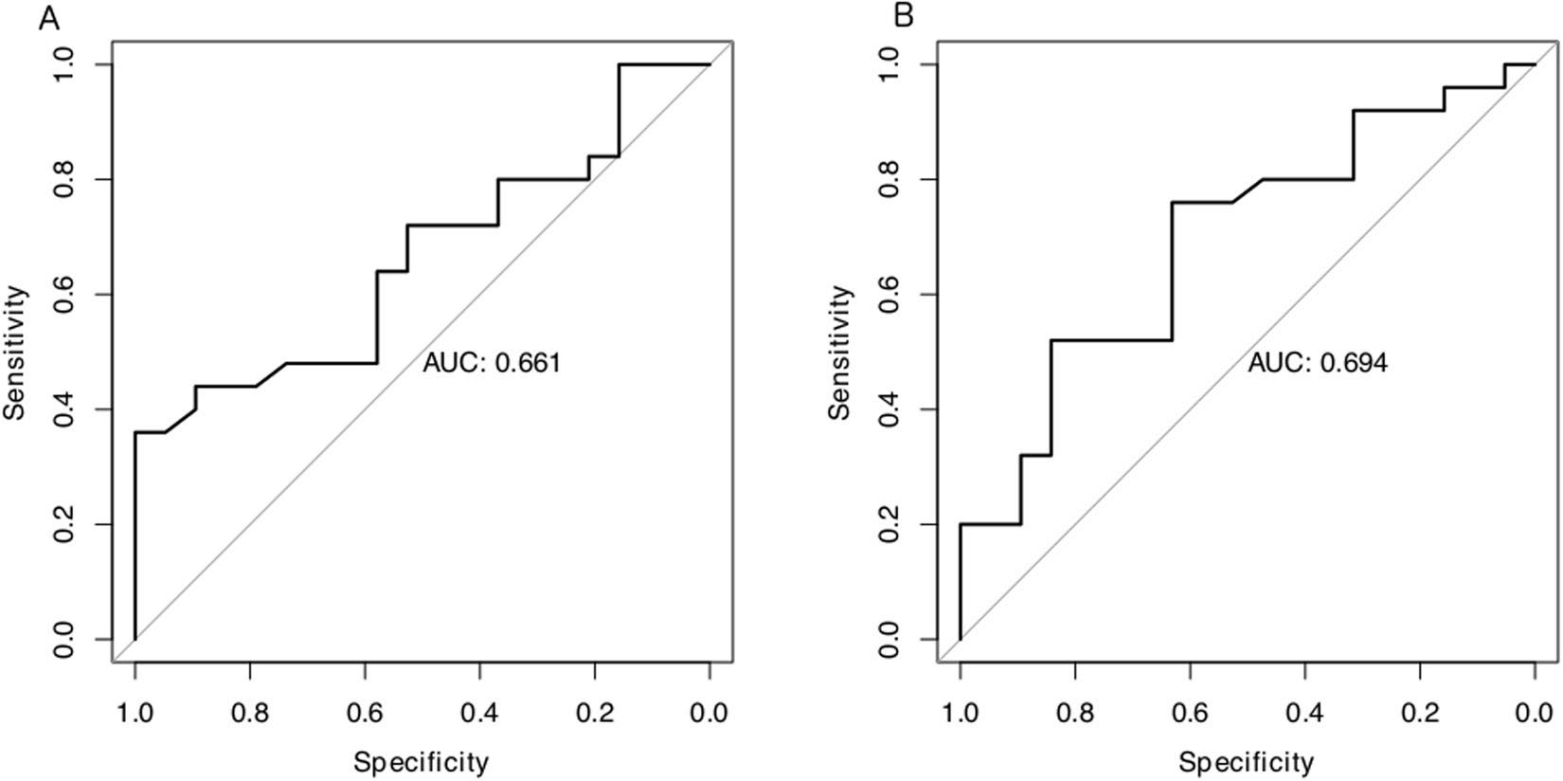
Receiver operator curve to predict HAPE susceptibility indicating the AUC for forced vital capacity (FVC) as panel A and single breath alveolar volume (SBVA) as panel B.

Pulmonary function testing has modest utility to discriminate HAPE susceptibility. To determine the usefulness of PFT parameters in predicting abnormal hypoxia-induced sPpa increase, we looked at the degree of correlation between the pulmonary function parameters found to be different in HAPE-S subjects and sPpa response to hypoxia (Fig. 1). Negative correlations were observed between SBVA or FVC, with baseline sPpa as well as hypoxia induced sPpa elevation (Table 4). Further, to determine their suitability as predictive markers, we also plotted receiver-operating characteristics (ROC) for these parameters (Fig. 2). None had a ROC area under curve greater than 0.8, when used individually. Even using a random forest technique based multi-parametric classification system, error rates remained close to 40%, comparable to the number of HAPE-S subjects with normal PFT.

**Table 4:** Pearson correlation coefficient values between systolic pulmonary artery pressure (sPpa) and pulmonary functions: forced vital capacity (FVC) and single breath alveolar volume (SBVA). (N = 25).

	FVC	SBVA
sPpa (Normoxia)	−0.23	−0.18
sPpa (Hypoxia)	−0.24	−0.29

## Discussion

Identification of otherwise fit subjects at risk of developing HAPE when taken to high altitude is an important problem. Others and we have previously reported a multitude of potential genetic or biochemical biomarkers^4–7^. However, there continues to be a need for simple nonspecialised physiological tests that can be done in resource-limited settings. While assessment of hemodynamic response during hypoxic challenge is a gold standard physiological test, it is neither simple, nor can it be performed outside specialised centres like ours. Receiver operator curve (ROC) curves represents excellent, good, and worthless tests plotted on the same graph. The accuracy of the test depends on how well the test separates the group being tested into those with and without the disease in question. Accuracy is measured by the area under the ROC curve (AUC).Here we show that low FVC is a potential marker of HAPE risk, but has poor sensitivity at acceptable levels of specificity (AUC = 0.66, sensitivity of about 40% at specificity of 80%), probably due to multiple etiological factors contributing to HAPE susceptibility like abnormal haemoglobin fractions as discussed in our previous study^7^. Additional PFT parameters beyond spirometry improve the discriminatory capacity somewhat, but about 40% of subjects who are HAPE susceptible may remain unidentified. Nevertheless, even modest predictive capacity of spirometry to identify HAPE-S subjects is potentially important since the test is easy to perform in any setting and is a good health screening tool in general. Using strict cutoffs that correctly classified all the HAPE-R subjects, about 20% of HAPE-S subjects could have been identified by FVC, which can be increased by simultaneous measurement of brain natriuretic peptide (BNP) showing AUC value 0.85, HIF1alpha and haemoglobin fractions^6,7^.Our findings are generally comparable to other reports, but with some important differences. HAPE-resistant subjects have previously been reported to have about 10% greater FVC compared to susceptible subjects in a small study^9^. Another study showed reduced FVC and TLC with no reduction in resting DLCO, but rebreathing DLCO was low at all times while doing normobaric and hypoxic exercise in HAPE-S^8^. We found no difference in FEV1/FVC and FRC in HAPE-S in contrast to previous studies^23^ however a sub group (HAPE-S2) showed low FVC, TLC and DLCO compared to control. Some of these differences may simply relate to different populations under study. Previous studies on susceptibility to HAPE were done on mountaineers and experimental protocols involved rapid ascent to high altitude. It is well known that physical exertion associated with rapid ascent is an independent factor for occurrence of HAPE. In contrast, our study is on Indian Army personnel who were low landers and deployed at high altitude on tenure basis. Strict instructions were followed to prevent high altitude maladies. Yet, despite these precautions during deployment, some of these personnel develop HAPE, presumably reflecting a greater susceptibility to HAPE than the mountaineers who developed HAPE during rapid ascent. This may explain why prior studies show comparable baseline sPpa between HAPE-S and HAPE-R subjects from a mountaineer population^24,25^ while we find higher baseline sPpa in HAPE-S subjects of this and previous studies^6,7^. Baseline difference in sPpa between two groups is partly related to inclusion criteria where subjects with normal pulmonary vascular response (normal PVR: sPpa < 38 mm Hg at Fio2 = i.e.0.12), apart from uneventful stay at high altitude, were included in HAPE-R group in this study^15^. However, HAPE-S was clinically defined since diagnosis was made on chest radiography and showed baseline raised BNP levels^6^ which in turn is inversely correlated with arterial oxygen levels thus the physiological differences remain meaningful.

The mechanistic basis of these observations remains unclear but there is strong clinical evidence linking FVC to vascular as well as pulmonary disease. John Hutchison, the inventor of spirometry, coined the term “vital capacity” i.e., capacity for life, as this seemed predictive for premature mortality^26^. This was subsequently confirmed by the Framingham Study where it was found to predict cardiovascular and all-cause mortality. It is worth mentioning here that FVC < 85% predicted has the highest hazard ratios for all cause mortality within framingham risk score groups^27^ which is consistent with present study showing FVC 84.83 ± 7.15% predicted in HAPE-S2 group. Since then additional studies have also confirmed associations between reduced FVC and a variety of non-pulmonary diseases including stroke, diabetes, hypertension, amongst others^28^. The chronic systemic inflammation^29^ and chronic hypoxia mediated vascular remodeling^6,7^ observed in our previous studies on non mountaineer HAPE-S can lead to a combination of small airway disease and reduced pulmonary vascular bed capacity, which may not manifest at sea level but prevent adequate compensatory increase in ventilation and perfusion, leading to HAPE. In support, the restrictive pattern shown by HAPE-S2 and asthma pattern^16^ shown by HAPE-S1 in our study has been strongly associated with presence of small airway or vascular disease in a large observational cross-sectional and longitudinal study^30^. Interestingly, interstitial lung diseases are an uncommon basis for such a pattern in otherwise healthy subjects from the general population. Surprisingly, raised baseline and increased endothelia-1(ET-1) levels at high altitude were reported in HAPE-S mountaineers^31^. ET-1 has been linked to lung fibrosis although ET-1 antagonist are ineffective in true fibrotic lung diseases like idiopathic pulmonary fibrosis, suggesting that ET-1 is part of a general lung remodeling pathway.

The major limitations of our study are that the subjects were all male and were studied after the development of HAPE. The incidence of HAPE is very low in army troops so prospective study was not feasible and the troops were all male. The possibility that the observed differences are a consequence of HAPE, rather than risk marker appears unlikely since it is well known that HAPE is non-inflammatory in nature and resolves quickly and completely with descent and/or oxygen^32^. Similar results have been shown in larger number of subjects studied earlier^33^. Thus it appears unlikely that the differences seen between HAPE-S and HAPE-R groups are a consequence of HAPE. Based on the total evidence available, it can be reasonably concluded that a restrictive pattern or an abnormally low vital capacity are predictive of increased HAPE risk. While the test characteristics are modest for FVC (AUC = 0.66) in the present study as expected due to multifactorial reasons for HAPE susceptibility however, its relevance is increased as it can be applied to both mountaineer and non mountaineer populations to predict HAPE susceptibility. Keeping in mind the ease and low-cost of performing spirometry, the potentially fatal course of HAPE and the huge logistical challenges of emergency transport to low altitude, this is a useful step towards personalised risk assessment.
